# The Evaluation of the Heavy Metal Leaching Behavior of MSWI-FA Added Alkali-Activated Materials Bricks by Using Different Leaching Test Methods

**DOI:** 10.3390/ijerph16071151

**Published:** 2019-03-30

**Authors:** Peng Xu, Qingliang Zhao, Wei Qiu, Yan Xue

**Affiliations:** 1 State Key Laboratory of Urban Water Resources and Environment, School of Environment, Harbin 150090, China; xupenghit@hit.edu.cn (P.X.); qlzhao@hit.edu.cn (Q.Z.); 2 Harbin Institute of Technology Environment Group CO., LTD, Harbin 150028, China; rockxue1227@sina.com

**Keywords:** municipal solid waste incineration fly ash, alkali activated, leaching test, pH-dependence

## Abstract

Alkali-activated materials (AAMs) not only have the potential to replace cement applications in architecture and civil engineering, but also have an excellent effect on the stabilization solidification of hazardous industrial wastes. This study used two types of municipal solid waste incineration fly ash (MSWI-FA)—grate firing fly ash (GFFA) and fluidized bed fly ash (FBFA)—as AAMs brick raw materials. It is discovered from this study that AAMs bricks with different weight ratios of GFFA and FBFA can both meet the required standard of GB21144-2007 (Solid concrete brick). From the results obtained from the four leaching tests, the equilibrium pH of the leachate varies, resulting in significant differences in the leaching of heavy metals in Raw GFFA, Raw FBFA, and AAMs bricks with GFFA and FBFA. The AAMs brick with the addition of GFFA and FBFA has an alkali activation system to encapsulate heavy metals. By comparing the results obtained from the CEN/TS 14429 leaching behavior test and the four batch leaching tests, it was found that the most influential factors for the heavy metal leaching concentration are whether the heavy metal has been solidified/stabilized in the samples. GFFA and FBFA tend to have consistent characteristics after being activated by alkali to form AAMs bricks. This can be confirmed by the acid neutralization ability concentrated on a specific pH range. The results obtained from CEN/TS14429 verified that the AAMs bricks with the addition of GFFA and FBFA have excellent environmental compatibility and that it provides a comprehensive evaluation on the environmental compatibility of the test materials and products. This demonstrated that the MSWI-FA is suitable for used as alkali-activated materials and its products have the potential to be commercially used in the future.

## 1. Introduction

Incineration is a common treatment used internationally for the disposal of municipal solid waste, advantaging a 90% volume reduction of raw wastes and thermal energy generation for electricity. It has the benefits of a short treatment time, less occupied space and few impacts on nearby lands, in comparison with direct buried treatment [1,2,3]. In China, the number of incinerating plants increased from 69 in 2006 to 898 in 2018, and they are expected to be responsible for over 10 million tons of waste in 2019, probably because incinerating municipal solid waste was recommended as one of the sources for electricity [4]. The hazardous solid wastes collected from air pollution preventing equipment, containing Dioxin [5], lead (Pb), chromium (Cr), cadmium (Cd), copper (Cu) and zinc (Zn) [6], have the risk of becoming secondary pollutants if municipal solid waste incineration (MSWI) fly ash (FA) is not properly treated. Therefore, many physical and chemical treatment systems are used for the solidification or stabilization of MSWI in China before transferring to landfill. However, the available landfills are rapidly reducing in China; it will soon to be a problem that requires urgent attention [1]. However, the leaching of soluble salts, heavy metals and other unstable intermediates from MSWI-FA limited the products’ application [7,8].

The resource technology of MSWI-FA is currently one of the alternatives for natural resource consumption, including the substitution of calcite with MSWI-FA to make environmentally-friendly cement [9]; or as a replacement of filler to be added to the concrete system [10]. The main purpose of these applications is to achieve energy saving and carbon emission reduction. In the LIFE+2008 project, ENV/IT/000434, MSWI-FA was used in industrial-scale applications by the Colloidal Silica Medium to Obtain Safe inert (COSMOS) technology. The products and technologies of this project have demonstrated stability and toxicity level that meet the regulatory standards. Related research and studies also showed that the polypropylene composites produced from MSWI-FA by COSMOS technology; its performance meets the standard requirements. Considering the factors of embodied energy and the CO_2_ footprint, MSWI-FA has considerable advantages to be selected as a substitution for natural materials [11]. The concept of “Turning waste into wealth” not only provides better control of the generation and whereabouts of the wastes, but also creates higher economic value. Currently, in China, MSWI-FA is an environmental problem that requires urgent attention. In order to protect the environment and achieve sustainable development, scholars and government authorities need to work together.

Alkali-activated materials (AMMs) have the potential to replace the traditional cement raw materials and most of the usable raw materials are industrial wastes such as furnace slag and fly ashes, etc. These materials have the advantages of low cost and low environmental load. Its mechanism and principle are summarized in the following steps: (1) mixing a high alkali agent containing rich silicon (Si) ions with inorganic materials containing CaO and Al_2_O_3_; (2) breaking the surface of the sodium silicate to release Ca^2+^ and Al^3+^; (3) reacting with Si ions to form hydration products [12]. The other is Si+Al system containing the furnace powder, which is used for the activation of SiO_2_ and CaO, and the hydration product is the calcium-silicate hydration gel having rich C-A-S-H and N-A-S-H compounds [13,14]. It can be noted from the reaction system that the chemical composition of MSWI-FA are mainly Ca, Si and Al [15,16]. It is, therefore, suitable to be used in an alkali activation system. Due to the fact that MSWI-FA contains hazardous components such as heavy metals, it classified as hazardous industrial wastes by the Chinese Government. To examine the feasibility of MSWI-FA to be used for alkali-activated materials (AAMs), the final brick products need to be further verified for environmental safety by the leaching test.

This research used two different types of MSWI fly ashes (MSWI-FA) to produce AAMs bricks: Grate Firing bed fly ash (GFFA) and Fluidized Bed fly ash (FBFA). In addition to the preliminary confirmation of AAMs bricks’ compressive strength, the leaching characteristics of MSWI-FA and AAMs bricks were evaluated by different leaching tests. This research also studied the environmental compatibility of AAMs bricks using four different leaching tests. The leaching test methods used in this research include China’s HJ/T 299-2007 [17] and HJ/T300-2007 [18] as well as the commonly used USEPA SW-846 Method 1311 (TCLP) [19] and USEPA SW-846 Method 1312 [20]. Additionally, CEN/TS 14429 [21] is considered as a batch leaching test which covers a wide range of acid-base environments. This research used CEN/TS 14429 to further study the heavy metal leaching behavior of AAMs bricks containing MSWI-FA under different environments.

## 2. Materials and Methods

### 2.1. Raw Materials for AAMs Brick

This study used two different types of municipal solid waste incineration fly ash (MSWI-FA) formed from different types of firing furnaces: Grate Firing bed fly ash (GFFA) and Fluidized Bed fly ash (FBFA). The GFFA and FBFA used in this study were obtained from the waste incineration plants in Shanghai and Anhui. The MSWI-FA were pre-treated by washing with de-ionized water (liquid/solid = 8:1) to reduce the influence of chloride on the performance of the AAMs brick [22].

The other raw materials used were coal fly ash (CFA) and alkali-activated reagent. The coal fly ash (F grade fly ash) was collected from a coal-fired power plant in Anhui and the Ground Granulated Blast-Furnace Slag (GGBFs) were purchased from the market. The alkali-activated reagent was prepared in-house which contained sodium silicate and sodium hydroxide. The weight ratio composition of purchased sodium silicate (Wenhua Chemical Co., Ltd., Philadelphia, PA, USA) was Na_2_O:SiO_2_:H_2_O = 8.20:29.7:62.1. The concentration of sodium hydroxide was 46% (*wt/wt*) and the molarity was 21.30 M. The chemical compositions of each raw material are shown in Table 1. CFA mainly consisted of Al_2_O_3_ and SiO_2_ [23]. GGBFs consisted of Al_2_O_3_, SiO_2_ and CaO [24]. GFFA consisted of CaO, with a high content of chloride salt (23.08%). The chloride content reduced to 5.42% after washing with de-ionized water. FBFA mainly consisted of CaO, SiO_2_, Al2O_3_ and the chloride content reduced to 0.86% after washing with de-ionized water. Table 1 showed that there is a substantial difference between the compositions of the two MSWI-FA. FBFA consists of SiO_2_ and CaO, and it contained 4.84% chloride salt, which is far lower than in GFFA. The heavy metal content of MSWI-FA is reported in Table 2. These two types of washed MSWI-FA contained various heavy metals, including Ba, Cu, Cr, Hg, Ni, Pb, Se and Zn.

### 2.2. Molding and Compressive Strength of AAMs Brick

#### 2.2.1. Design of Mix Proportion

This study used the molding procedure to produce AAMs bricks. The materials used for AAMs bricks and mix proportions are shown in Table 3. The density of the bricks was 1850 kg/m^3^ with the different mix proportions. The mix proportions were designed based on the results of previous studies. The module ratio of SiO_2_/Na_2_O in the alkali-activated reagent was controlled at 1.20. The quantity of alkali-activated reagent to be added was calculated based on the Na_2_O content in the GGBFs (3.75% w.t. Na_2_O).

#### 2.2.2. Molding Process

The instructions for the molding process are as follows. Weigh the alkali-activated reagent and CFA according to the designed mix proportions. Well-mix the alkali-activated reagent and CFA and let the mixture sit for 10 min. Add GGBFs and MSWI-FA (GFFA or FBFA) and mix. Add an appropriate amount of water during the mixing to obtain a wet powder. Place the well-mixed wet powder into a mold of 20 cm × 10 cm × 5 cm. Set the pressure to 100 kg/cm^2^ and start the molding. Once the molding is completed, immediately release the brick from the mold and place it under room temperature (25 °C) for one day. Subsequently, soak the brick in saturated lime water to proceed with the curing.

#### 2.2.3. Compressive Strength Test

In order to determine the compressive strength development of the alkali-activated bricks, this study performed compressive strength tests on alkali-activated bricks cured for 3 days, 7 days, 14 days and 28 days. The compressive strength test was conducted according to the GB standard methods (GB 21144-2007). The stacked bricks, 40 mm to 90 mm thick, were used to measure the maximum amount of compressive load on the bearing area of each brick using the compressive mechanism (ADR Touch Control Pro 3000 BS, ELE, UK).

### 2.3. Leaching Test of Heavy Metals

MSWI-FA contained heavy metals and other hazardous materials, thus classified as hazardous industrial wastes. This research studied AMMs bricks produced from MSWI-FA and other industrial wastes. Other than meeting the compressive strength stated in the regulation standards, the environmental safety of the AMMs bricks also needs to be verified by the leaching tests.

This study compared the leaching tests commonly used in China: HJ/T 299-2007 (Solid Waste-Extraction Procedure for the Leaching Toxicity-Sulfuric Acid and Nitric Acid Method) and HJ/T 300-2007 (Solid Waste-Extraction Procedure for the Leaching Toxicity-Acetic Acid Buffer Solution Method), with leaching tests widely used in the world: 1311 TCLP and 1312 Synthetic Precipitation Leaching Procedure of USEPA SW-846. Additionally, CEN/TS 14429 (characterization of waste. Leaching behavior tests. Influence of pH on leaching with initial acid/base addition) was also used in this study. This test procedure added different concentrations of acid or base to reach equilibrium; the formed leachates were subsequently analyzed for the heavy metal concentration. 

The major difference between the test method in CEN/TS 14429 and batch leaching testing methods such as HJ/T 299-2007, HJ/T 300-2007, TCLP and the Synthetic Precipitation Leaching Procedure was that the test method in CEN/TS 14429 demonstrated the heavy metal concentration of the leachates under different pH values with acid or base reactions. The equilibrium pH value range was 4–12. The acid-base neutralization curve can be drawn according to the test samples and the amount of equivalent concentrations of the acid or base to be added. The curve can be used to assess the tolerance of the test samples to the acid/base in the environment. By testing the sample with the test method in CEN/TS 14429, the heavy metals concentration of the leachate at each equilibrium pH can be assessed. It is speculated that the test method in CEN/TS 14429 has the potential to predict the results of HJ/T 299-2007, HJ/T 300-2007, TCLP and Synthetic Precipitation Leaching Procedure. 

The leachate was filtered and the heavy metal concentrations of Ba, Cr, Cu, Pb and Zn were analyzed by ICP-OES (Perkin-Elmer 2100 DV, Wellesley, USA). In order to facilitate the clarification of the differences between the aforementioned leaching test methods, the leaching test methods were summarized in Table 4.

#### 2.3.1. HJ/T 299-2007 Solid Waste-Extraction Procedure for Leaching Toxicity—The Sulfuric acid and Nitric Acid Method

HJ/T 299-2007 was used to examine whether the waste would leach in an acid rain environment, causing environmental damage. The test procedure is summarized as follows:

1. Sieve the dried sample through a 9.5 mm sieve mesh.
2. Prepare the simulated acid rain by adding a 2:1 weight percent mixture of sulfuric and nitric acids to water until the pH is 3.20 ± 0.05.
3. Weigh an approximately 150–200 g sample into a 2 L PE bottle. Then add simulated acid rain at 10 times the sample weight.
4. Seal the PE bottle and place it in a rotary agitator; rotate at 30 ± 2 rpm for 18 h ± 2 h at 23 ± 2 °C.
5. Filtrate the leachate with a 0.6–0.8 μm membrane filter. The concentration of all metals in the leachate was analyzed using ICP-OES.

#### 2.3.2. HJ/T 300-2007 Solid Waste-Extraction Procedure for Leaching Toxicity—The Acetic Acid Buffer Solution Method

HJ/T 300-2007 simulated the condition of waste berried in the sanitary landfill and examine whether the waste formed humic acid (e.g., acetic acid) and leach into the surrounding environment causing damages. The test procedure is summarized as follows:

1. Sieve the dried sample through a 9.5 mm sieve mesh.
2. Dilute 17.25 mL of glacial CH_3_CH_2_OOH with reagent-grade water to a volume of 1 liter. The pH of the reagent should be 2.64 ± 0.05.
3. Weigh an approximately 75–100 g sample into a 2 L PE bottle. Then add acetic acid at 20 times the sample weight.
4. Seal the PE bottle and place in a rotary agitator; rotated at 30 ± 2 rpm for 18 h ± 2 h at 23 ± 2 °C.
5. Filtrate the leachate with a 0.6–0.8 μm membrane filter. The concentration of all metals in the leachate was analyzed using ICP-OES.

#### 2.3.3. USEPA SW-846 Methods 1311 Toxicity Characteristic Leaching Procedure

TCLP is similar to HJ/T 300-2007, it also simulated the condition of waste buried in the sanitary landfill and examined whether the waste forms humic acid (e.g., acetic acid) and leach into the surrounding environment causing damages. The major difference was the amount of acetic acid used in each method. TCLP used only 1/3 the amount of acetic acid than used in HJ/T 300-2007. The test procedure is summarized as follows:

1. Sieve the dried sample through a 9.5 mm sieve mesh.
2. Dilute 5.7 mL of glacial CH_3_COOH with reagent-grade water to a volume of 1 L. The pH value of the reagent should be 2.88 ± 0.05.
3. Weigh an approximately 75–100 g sample into a 2 L PE bottle. Then add acetic acid at 20 times the sample weight.
4. Seal the PE bottle and place it in a rotary agitator; rotated at 30 ± 2 rpm for 18 h ± 2 h at 23 ± 2 °C.
5. Filtrate the leachate with a 0.6–0.8 μm membrane filter. The concentration of all metals in the leachate was analyzed using ICP-OES.

#### 2.3.4. USEPA SW-846 Methods 1312 Synthetic Precipitation Leaching Procedure

USEPA SW-846 Methods 1312 simulated the acid rain environment to examine whether the waste would leach and damage the environment. The acidity of the acid rain was simulated based on the acid rain collected from the area of east Mississippi River. The test procedure is almost the same as HJ/T 299-2007, but parameters such as the ratio of sulfuric acid and nitric acid, the pH of the reagent, and the controlled liquid-solid ratio are different. The test procedure is summarized as follows:

1. Sieve the dried sample through a 9.5 mm sieve mesh.
2. Prepare the simulated acid rain by adding a 3:2 weight percent mixture of sulfuric and nitric acids to water until the pH is 4.20 ± 0.05.
3. Weigh an approximately 75–100 g sample into a 2 L PE bottle. Then add simulated acid rain at 20 times the sample weight.
4. Seal the PE bottle and place it in a rotary agitator; rotated at 30 ± 2 rpm for 18 h ± 2 h at 23 ± 2 °C.
5. Filtrate the leachate with a 0.6–0.8 μm membrane filter. The concentration of all metals in the leachate was analyzed using ICP-OES.

#### 2.3.5. CEN/TS 14429 Characterization of Waste—The Leaching Behaviour Test—The Influence of pH on Leaching with Initial Acid/Base Addition

1. Sieve the dried sample through a 1 mm sieve mesh.
2. Prepare the reagent by diluting the acid/alkali solution to the respective equivalent concentration required.
3. Weigh a 15 g sample into a 250 mL PE bottle. Then add the reagent at a weight/volume ratio of 1/10.
4. Seal the PE bottle and place it in a rotary agitator; rotated at 10 rpm at 23 ± 2 °C.
5. Measure the pH at 4 h, 44 h, and 48 h, respectively. The pH value at 48 h and at 44 h should be less than 0.3 to confirm that the equilibrium condition is reached.
6. Collect leachates with an equilibrium pH of 4–12, and the pH interval between the samples has to be less than 1.5.
7. Filtrate each leachate with a 0.45 μm membrane filter. The concentration of all metals in the leachate was analyzed using ICP-OES.

## 3. Results and Discussion

### 3.1. Compressive Strength of AAMs Bricks with Different Typed MSWI Fly Ashes

This study used two different types of MSWI fly ashes (MSWI-FA) formed from different types of firing furnace: Grate Firing bed fly ash (GFFA) and Fluidized Bed fly ash (FBFA). Both MSWI-FA were pre-treated by washing with de-ionized water to reduce the soluble chloride salt, and then mixed with GGBFs, CFA and alkali-activating reagent to produce AAMs bricks. Various weight ratios of 10%, 20%, 30% MSWI-FA were added and mixed with CFA and GGBFs to produce AAMs bricks.

#### 3.1.1. The Effects of GFFA Addition on the Characteristics of AAMs Bricks

**Figure 1:**
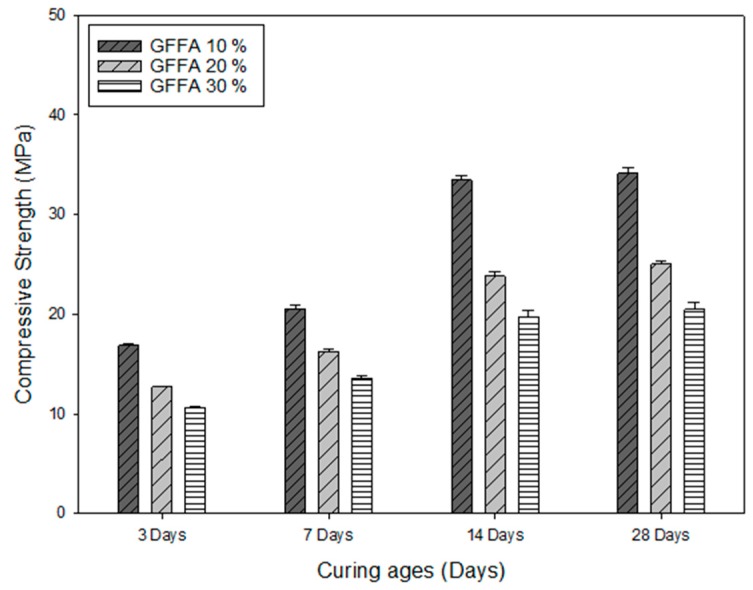
The compressive strength of different percentages of the grate firing fly ash (GFFA) in the alkali-activated material (AAM) bricks.

The main component of GFFA was calcium oxide (CaO). The soluble chloride salt can be reduced from 23.08% to 5.42% by washing pretreatment. To investigate the compressive strength of the GFFA containing AAMs bricks, different percentages corresponding to 10%, 20% and 30% of GFFA were added and the samples were tested at 3-, 7-, 14- and 28-day curing periods. Figure 1 demonstrated that at 28-days of curing age, the compressive strengths of 34.16 MPa, 25.05 MPa, and 20.46 MPa were respectively reported with the addition of 10%, 20% and 30% GFFA into the AAMs bricks. A decrease in the compressive strength of the bricks was observed by increasing the amount of the added GFFA, indicating that a large amount of Ca in GFFA had a negative effect on the alkali-activated system. It was found in alkali-activation related researches that an excessive amount of CaO will lead to decreased efficiency of the alkali-activation system and result in an incomplete hydration reaction, which further affects strength development in the slurry [15]. In addition, the literature also indicated that excessive Ca(OH)_2_ in the alkali-activated or Geopolymer system has a negative impact on the development of compressive strength [25]. One of the probable reasons was that parts of chloride salts dissolved in the water during curing, forming many pores in the bricks to reduce the strength of the bricks. Excessive chloride salts may cause physical defects such as pores in the alkali-activated material, which damages its microstructure and affect the compressive strength [22].

#### 3.1.2. The Effects of FBFA Addition on the Characteristics of AAMs Bricks

**Figure 2:**
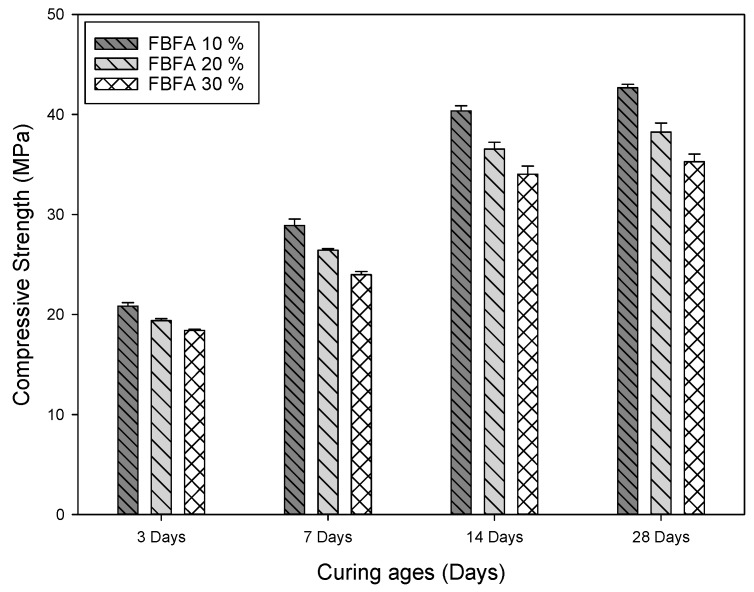
The compressive strength of different percentages of the fluidized bed fly ash (FBFA) in the AAMs bricks.

The FBFA contains high aluminum and silicon produced from the addition of quartz sand or slack, which were used to facilitate tcomplete combustion of the municipal waste in the fluidized bed [26]. The soluble chloride salts removal was achieved by washing pretreatment; the chloride salt content was reduced from 4.84% to 0.86%. It is known that the level of chloride salt in the FBFA is lower than the GFFA. To investigate the compressive strength of the FBFA containing AAMs bricks, different percentages of 10%, 20% and 30% of FBFA were added and the samples were tested at 3-, 7-, 14- and 28-days curing periods. Figure 2 illustrated that the compressive strengths of 42.68 MPa, 38.25 MPa, and 35.27 MPa were respectively reported with the addition of 10%, 20% and 30% FBFA to the AAMs bricks.

China’s common brick standard for solid concrete brick (GB 21144-2007) stated that the 28-days curing age bricks must have a minimum compressive strength of 15 MPa. After washing pretreatment, the dechlorinated GFFA and FBFA were used to produce AAMs bricks. The compressive strength of AAMs bricks added with the 30% weight ratio of GFFA and FBFA can meet the standard requirement of GB 21144-2007. This confirms that AAMs brick technology has the potential to be applied for incineration fly ash recycling and reuse.

The difference between the compositions of GFFA and FBFA reflects on the AAMs bricks’ compressive strength and properties. Comparing to GFFA, FBFA was more suitable to be used as raw materials for AAMs bricks. The reason is that the composition of FBFA has a higher level of CaO, SiO_2_ and Al_2_O_3_ than GFFA and it can be determined from the related literature that the ratio of CaO, SiO_2_ and Al_2_O_3_ in the AAMs system affects the strength of the recycled products [27].

### 3.2. Leaching Test of Two Types AAMs Bricks

The municipal solid waste incineration fly ash (MSWI-FA) was classified as hazardous waste, thus, different leaching tests were used to test the heavy metals solidification and stabilization in the MSWI-FA containing AAMs bricks. To examine the environmental compatibility of original fly ash and the AMMs, bricks with 10%, 20%, 30% MSWI-FA were added. This study used four leaching test methods commonly used in China and the United States, and the leachate were analyzed for five targeted heavy metals: copper (Cu), zinc (Zn), lead (Pb), chromium (Cr), barium (Ba).

#### 3.2.1. Leaching Test Results of the Two Types of AAMs Bricks by HJ/T 299-2007 (Solid Waste-Extraction Procedure for Leaching Toxicity-Sulfuric Acid and Nitric Acid Method)

GFFA, FBFA, and MSWI-FAs containing AAMs bricks were tested by HJ/T 299-2007. The concentration of Cu, Zn, Pb, Cr and Ba in the leachates were analyzed and reported in Table 5. Comparing the results of MSWI-FA and MSWI-FAs containing AAMs bricks, it was found that MSWI-FA containing AAMs bricks efficiently inhibited the leaching of heavy metals. The heavy metal content in AAMs bricks leachate was much lower than that in the raw MSWI-FAs. As the amount of MSWI-FA increases, the content of the heavy metals in the leachate also increases. The concentration of Pb in the leachate is close to the regulation limit set in the identification standards for hazardous wastes—identification for extraction toxicity (GB 5085.3-2007). The heavy metal content in the leachate of AAMs bricks is well below the regulation limits. Upon the completion of the leaching test, the pH value of the leachate is between 11.9 and 12.3, which is highly alkaline. It can be concluded that amphoteric elements such as Zn, Pb and Cr have higher leachability [28].

#### 3.2.2. The Leaching Test Results of the Two Types of AAMs Bricks by HJ/T 300-2007 (The Solid Waste-Extraction Procedure for Leaching Toxicity-Acetic Acid Buffer Solution Method)

It is discovered from the results of HJ/T 300-2007 (Table 6) that the raw MSWI-FA’s leaching of Pb exceeded the limits set in GB 16889-2008 (standard for pollution control on the landfill site of municipal solid waste). Due to the heavy metal encapsulation of the alkali-activated system, the leaching of Pb from AMMs bricks was below the regulation standard limit. After analyzing the test results, the pH of GFFA leachate was alkaline, whereas the pH of FBFA leachate was acidic. The pH revealed that excessive heavy metals were leaching into the leachate. However, the pH values of AAMs bricks were between 6 and 6.7, the leaching of heavy metals showed a similar pattern. It can be concluded that the bricks belong to AAMs and the final pH value of the leachates was similar.

#### 3.2.3. The Leaching Test Results of the Two Types of AAMs Bricks by the USEPA SW-846 Method 13110 (Toxicity Characteristic Leaching Procedure)

USEPA SW-846 Method 1311: Toxicity characteristic leaching procedure (TCLP) is a common leaching test used internationally. This procedure was designed to determine whether the tested sample is environmentally hazardous. The USEPA SW-846 Method 1311 is similar to the HJ/T 300-2007 leaching test method in China, except that the acetic acid amount of the reagent used in HJ/T 300-2007B is three times greater than that of reagent B used in the USEPA SW-846 Method 1311.

Table 7 reported the results of SW-846 Method 1311 TCLP. It is noticed that GFFA showed leaching of five heavy metals. The AAMs bricks containing 20% and 30% of GFFA showed the leaching of Pb and Ba. Only FBFA and FBFA containing AAMs bricks showed the leaching of Ba. The results were quite different from those of HJ/T 299-2007 and HJ/T 300-2007. In addition, the equilibrium pH values of the GFFA and FBFA leachates were 12.14 and 8.54, respectively.

The results showed that the limitation of encapsulation in alkali-activation technology affected AAM’s ability to inhibit the leaching of heavy metals. Additionally, the concentration of acetic acid used in HJ/T 300-2007 and TCLP was different, leading to the difference of the equilibrium pH values of the AAMs brick leachates (pH = 10.13–10.56). Therefore, the final pH value of the leachates will also affect the amount of heavy metal leaching from the test samples [29].

#### 3.2.4. The Leaching Test Results of the Two Types of AAMs Bricks by the USEPA SW-846 Method 1312 (Synthetic Precipitation Leaching Procedure)

USEPA SW-846 Method 1312: Synthetic Precipitation Leaching Procedure is designed to determine the mobility of both organic and inorganic samples present in liquids, soils, and wastes. This method is used to evaluate the releasing capacity of the harmful substance of the test sample in an acid rain environment, so no limit was defined. 

This method was similar to HJ/T 299-2007 as both methods were used to test leach the samples in an acid rain environment. It was discovered from the leaching test results shown in Table 8 that the acids and their concentrations used in the method were similar to that of HJ/T 299-2007. Therefore, the final equilibrium pH value (pH = 11.94–12.28) and the amount of heavy metal leaching was also similar to the results of HJ/T 299-2007. Although the results were similar, it was observed that the leaching amount in USEPA SW-846 Method 1312 was slightly lower. This resulted from the difference of the liquid/solid ratio; the liquid/solid ratio of USEPA SW-846 Method 1312 was 1/20 whereas the liquid/solid ratio of HJ/T 299-2007 was 1/10.

Likewise, due to the limitation of encapsulation, the leaching amount of AAMs bricks was much lower than that of raw MSWI-FA.

### 3.3. The Evaluation of the Leaching Behavior of the Two Types of AAMs Bricks by CEN/TS 14429

HJ/T 299-2007, HJ/T 300-2007, the USEPA SW-846 method 1311 and the USEPA SW-846 method 1312 only have one equilibrium pH value; however, the equilibrium pH values of the leachate obtained from different batch leaching tests were different, which led to the difference between the heavy metal concentrations of the leachates. Therefore, CEN/TS 14429 covered a wide range of acid-base environments to examine the heavy metal leaching behavior of the test samples at different pH values [30].

#### 3.3.1. Acid Neutralizing Capacity of Raw MSWI-FAs and MSWI-FA Containing AAMs Bricks

**Figure 3:**
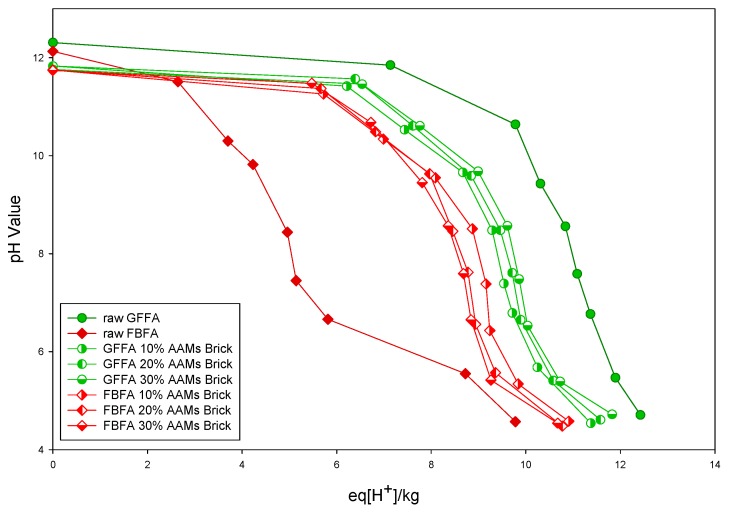
The acid neutralizing capability curves of raw GFFA/FBFA and municipal solid waste incineration fly ask (MSWI-FA) containing AAMs bricks.

Figure 3 displayed the acid neutralizing capability curves of raw GFFA, raw FBFA and GFFA- and FBFA-containing AAMs bricks. It can be observed from Figure 3 that the pH value of raw GFFA is higher than raw FBFA, and also its acid neutralizing capacity was significantly greater than raw FBFA. It can be inferred that GFFA contained a large amount of alkaline substances, such as calcium oxide and calcium hydroxide. In addition, the particle size of GFFA was smaller than that of FBFA. From the curve of GFFA- and FBFA-containing AAMs bricks, the alkalinity increased with the increasing GFFA amount, while the increasing FBFA amount decreases the alkalinity of the AAMs bricks.

It can be observed from the acid neutralizing capability curves of MSWI-FA-containing AAMs bricks that GFFA-containing AAMs bricks still exhibit a greater acid neutralizing capacity than FBFA-containing AAMs bricks, but the difference is less obvious than the ones seen in raw MSWI-FA. This could be caused by the use of high alkaline alkali-activating reagents in the production of AAMs bricks converting the MSWI-FA, CFA and GGBFs to AAMs, resulting in the similar acid neutralizing ability of AAMs bricks.

#### 3.3.2. Leaching Behavior of Heavy Metals from Raw MSWI-FA and MSWI-FA Containing AAMs Bricks at Different pH Values

**Figure 4:**
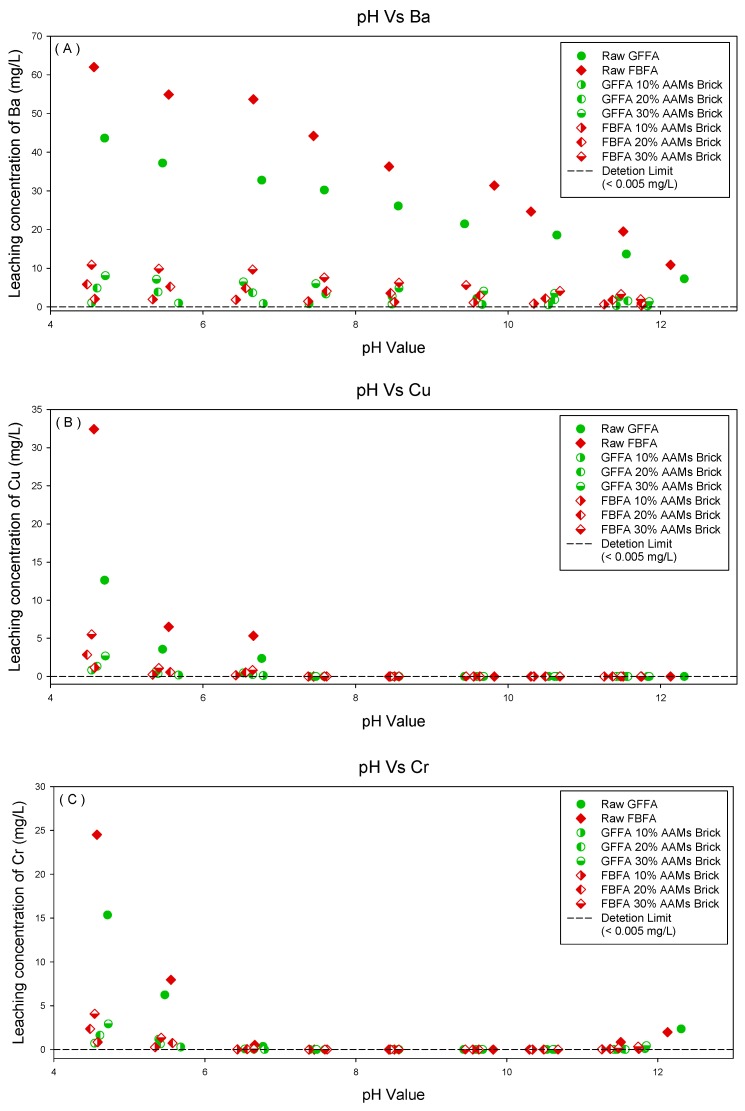

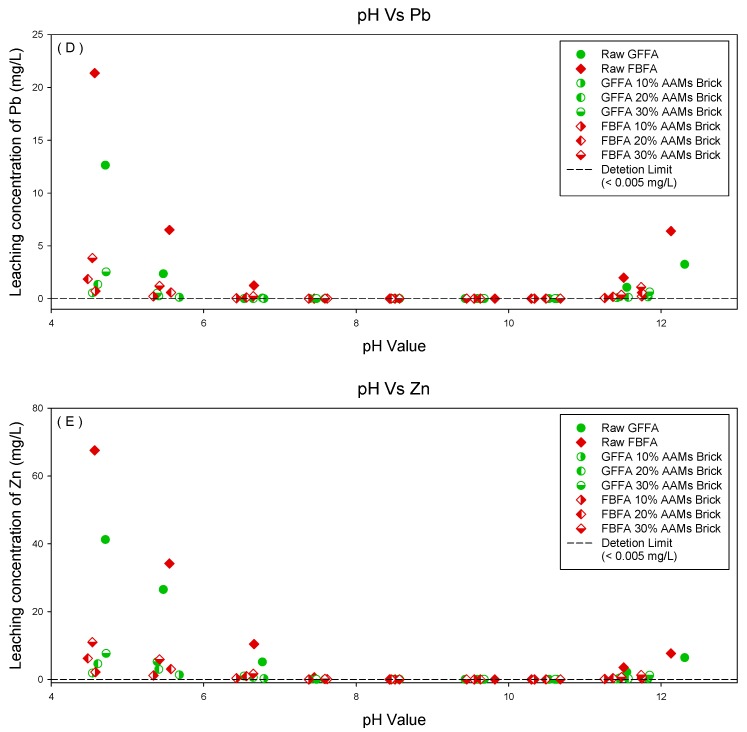
The results of raw GFFA, raw FBFA and MSWI-FA containing AAMs bricks using the leaching test in CEN/TS 14429. (**A**) Ba; (**B**) Cu; (**C**) Cr; (**D**) Pb; (**E**) Zn.

Figure 4A–E demonstrates the leaching behavior of the heavy metals (Ba, Cu, Cr, Pb and Zn) from raw GFFA, raw FBFA and MSWI-FA containing AAMs bricks under different equilibrium pH conditions. The Y-axis in Figure 4A–E represents the concentrations of the heavy metals respectively. It was observed from Figure 4A–E that the leaching of Cu from the samples only occurred in an acidic environment [31]. The leaching of Cu, Pb, Zn were observed in both acidic environments (pH < 6) and alkaline environments (pH > 12) because of their amphoterism [32]. The solubility of Ba salts in water was greater than that of the aforementioned heavy metals, thus, the leaching of Ba decreased as the equilibrium pH increases [33]. 

By comparing the results of raw GFFA and raw FBFA, it was discovered that the heavy metal concentration of FBFA leachate was significantly higher than that of GFFA within the same pH range under acidic environments because the heavy metals content of FBFA was greater than that of GFFA. Therefore, under the same acidic pH environment, the content of heavy metals in MSWI-FA directly affected the concentration of heavy metals in the leachates. A similar situation can also be observed from the results of GFFA- and FBFA-containing AAMs bricks. Even though the leaching of heavy metals can be effectively inhibited by MSWI-FA containing AAMs bricks, the leaching of heavy metals from the AAMs bricks within the same pH range also increased with the amount of MSWI-FA. Moreover, under the same test conditions, the heavy metals leaching from FBFA-containing AMMs bricks was higher than that from GFFA-containing AAMs bricks. 

Summarized from the above leaching behavior, it can be observed that GFFA and FBFA have similar material properties after being made into AAM bricks by alkali-activation technology and the acid neutralizing ability of the AAMs bricks was concentrated within a specific pH range. It was known that the environmental compatibility of AAMs and the heavy metal leaching behavior can also be examined by the leaching test in CEN/TS 14429. The results showed that the leaching of a large amount of hazardous heavy metals (Cu, Cr, Pb, etc.) only occurred with a pH below 6, indicated that the GFFA- and FBFA-containing AAMs bricks have good environmental compatibility and safety.

#### 3.3.3. The Comparison of the Results Obtained from the Test Method in CEN/TS 14429 and the Other Four Leaching Tests

Section 3.1 and Section 3.2 used four leaching tests to verify AAMs bricks’ environmental compatibility. The above-mentioned leaching tests used different types of acid reagent and concentrations to prepare the leaching reagents. After the tests, the different leachates only had one equilibrium pH value. However, the CEN/TS 14429 covers a wide range of acid-base environment (pH = 4–12) and comprehensively examines the correlation between the pH values and heavy metal leaching behavior of the raw GFFA, raw FBFA and MSWI-FA containing AAMs bricks.

Among the four leaching tests of raw GFFA and raw FBFA, the results of the HJ/T 299-2007 and USEPA SW-846 Method 1312 were similar and the equilibrium pH of the leachates was in between 12.13 and 12.29. The respective equilibrium pH values of the leachates in HJ/T 300-2007 were 12.04 and 5.48. The equilibrium pH values of the leachates in USEPA SW-846 Method 111 were 12.14 and 8.54, respectively. The heavy metal leaching results obtained from CEN/TS 14429 corresponded to the aforementioned leaching tests. In the test of the equilibrium pH values of the GFFA- and FBFA-containing AAMs bricks, the results of HJ/T 299-2007 were concentrated at pH values between 11.9 and 12.2; the results of HJ/T 300-2007 were concentrated at pH values in the range of 6–6.7; the results of USEPA SW-846 Method 1311 were concentrated at pH values between 10.1 and 10.6; the results of USEPA SW-846 Method 1312 were concentrated at pH values between 11.9 and 12.2. It was observed that the equilibrium pH value of the leachate was concentrated in a certain pH range, which indicated that the properties of the AAMs bricks tend to be consistent by the addition of GFFA or FBFA and the alkali-activating process. On the other hand, influenced by the limitation of encapsulation in the alkali activation technology, heavy metals can be properly stabilized in the bricks.

From the above information, it can be concluded that for the evaluation of recycled raw materials or products made from hazardous industrial wastes, CEN/TS14429 can be applied to cover the common batch leaching tests used in various countries. It provides a more comprehensive assessment of the heavy metal leaching behavior of the test materials or products under different environmental pH conditions.

## 4. Conclusions

In China, the MSWI-FA can be divided into grate firing fly ash (GFFA) and fluidized bed fly ash (FBFA) according to the types of the furnace used. As the amount of Ca, Si, Al, Cl and leaching behavior in GFFA and FBFA have a significant difference, they should be defined as different recycling materials.

In this research, weight ratios of 10%, 20%, and 30% GFFA or FBFA were added to the alkali-activation system and the compressive strength was obtained from the 28-day curing AAMs bricks. The compressive strength of FBFA containing AAMs bricks was 35.27 MPa–42.68 MPa, which was higher than that of the GFFA containing AAMs bricks (20.46 MPa–34.16 MPa). With MSWI-FA reused as raw materials for AAMs bricks, in terms of the engineering property requirement, FBFA has better performance than GFFA. Based on the results of compressive strength, both AAMs bricks conformed with the Chinese regulation standard (GB-21144-2007).

The alkali activation technology effectively inhibited the heavy metal leaching from fly ash, given good environmental compatibility to the AAMs bricks. The equilibrium pH values of the leachate obtained from different batch leaching tests were different, which led to the difference between the heavy metal concentrations of the leachates. Comparing the CEN/TS 14429 leaching test with other four leaching tests (HJ/T 299-2007, HJ/T 300-2007, USEPA SW-846 Method 1331, USEPA SW-846 Method 1312), we noticed that CEN/TS 14429 covered a wide range of acid-base environments to examine the heavy metal leaching behavior of the test samples at different pH values. By comparing the leaching test results of raw GFFA, raw FBFA and MSWI-FA containing AAMs bricks, it was discovered that the amount of heavy metal leaching has a weak correlation to the acid solution used or the liquid-solid ratio specified in the method. The GFFA or FBFA containing AAMs bricks produced by alkali-activation tend to have more consistent properties and the acid neutralizing ability of the AAMs bricks was concentrated in a specific range. It can be observed from the results of heavy metal leaching that it is only possible to have excessive leaching of hazardous heavy metals such as Cu, Cr, Pb, etc., at pH values below 6. The presence of amphoteric elements such as Pb and Cr caused leaching in highly alkaline environments. The results of CEN/TS14429 proved that the GFFA- and FBFA-containing AAMs bricks have good environmental compatibility and safety.

Therefore, the properties of GFFA- and FBFA-containing AAMs bricks were considered as recycling products by adjusting the different weight ratios of fly ashes. They can meet the required engineering properties and environmental safety standards and can be used in the application of civil and construction engineering.

## Figures and Tables

**Table 1:** The chemical compositions of raw materials (*wt*%).

Chemical Composition	Na_2_O	MgO	Al_2_O_3_	SiO_2_	CaO	Fe_2_O_3_	Cl
CFA	0.26	0.82	24.38	56.83	5.90	5.95	-
GGBFs	-	5.84	14.77	32.38	42.78	0.35	-
Sodium silicate	8.20	-	-	29.7	-	-	-
Sodium hydroxide	46	-	-	-	-	-	-
GFFA	2.7	1.51	0.82	3.77	63.34	1.12	23.08 (5.42)
FBFA	0.29	4.62	11.18	22.67	41.95	7.48	4.84 (0.86)

CFA: coal fly ash; GGBFs: Ground Granulated Blast-Furnace Slag; GFFA: Grate Firing bed fly ash; FBFA: Fluidized Bed fly ash.

**Table 2:** The content of heavy metal in Grate Firing bed fly ash (GFFA) and Fluidized Bed fly ash (FBFA) (mg/kg).

Heavy Metal	Ba	Cu	Cr	Hg	Ni	Pb	Se	Zn
GFFA	332	4518	824	37	381	67	64	3045
FBFA	485	5861	754	34	348	864	62	4256

**Table 3:** The mix proportion design of municipal solid waste incineration fly ash (MSWI-FA) containing alkali-activated bricks.

Sample ID	MSWI-FA (kg/m^3^)	CFA (kg/m^3^)	GGBFs (kg/m^3^)	Alkali-Activated Reagent (g)	
				NaOH	Na_2_SiO_3_
GFFA 10%	370	740	555	64.7	135.3
GFFA 20%	555	555	555		
GFFA 30%	740	370	555		
FBFA 10%	370	740	555		
FBFA 20%	555	555	555		
FBFA 30%	740	370	555		

**Table 4:** The comparison table of different leaching test methods used in this study.

Country	China		USA		European Commission
Method no.	HJ/T 299-2007	HJ/T 300-2007	US EPA SW-846 Methods 1311	US EPA SW-846 Methods 1312	CEN/TS 14429
Design concept	Batch; single	Batch; single	Batch; single	Batch; single	Batch; multiple
Sample size	Less than 9.5 mm	Less than 9.5 mm	Less than 9.5 mm	Less than 9.5 mm	Less than 1 mm
S/L ratio	1:10	1:20	1:20	1:20	1:10
Acid used in the reagent	sulfuric and nitric acids (Dilute to specified pH value)	Glacial acetic acid (17.25 mL/L)	Glacial acetic acid (5.7 mL/L)	sulfuric and nitric acids (Dilute to specified pH value)	nitric acids (Adjust the concentration according to the sample condition)
pH of the reagent	3.20 ± 0.05	2.64 ± 0.05	2.88 ± 0.05	4.20 ± 0.05	The pH of the reagent varies depending on the sample
Reaction time	18 ± 2 h	18 ± 2 h	18 ± 2 h	18 ± 2 h	48 h

**Table 5:** The results of the leaching test using the analytical method HJ/T 299-2007.

MSWI-FA Type	Added Amount	pH Value	Heavy Metal				
			Cu	Zn	Pb	Cr	Ba
			Unit (mg/L)				
GFFA	Raw	12.28	0.184	5.47	3.51	1.84	6.84
	10%	12.12	0.0019	0.089	0.12	0.034	0.012
	20%	12.14	0.0039	0.243	0.14	0.051	0.021
	30%	12.18	0.0050	0.465	0.21	0.062	0.054
FBFA	Raw	12.13	0.743	6.84	4.47	1.24	8.25
	10%	11.94	0.059	0.037	0.51	0.018	0.0019
	20%	11.96	0.124	0.069	0.62	0.046	0.0029
	30%	11.94	0.236	0.085	0.72	0.052	0.0041
GB5085.3-2007			100	100	5	15	100

ND: < 0.2 × 10^−3^ (mg/L).

**Table 6:** The results of the leaching test using analytical method HJ/T 300-2007.

MSWI-FA Type	Amount of Addition	pH Value	Heavy Metal				
			Cu	Zn	Pb	Cr	Ba
			Unit (mg/L)				
GFFA	Raw	12.04	0.172	3.54	2.68	1.38	5.26
	10%	6.47	0.037	1.84	0.03	0.84	2.92
	20%	6.51	0.081	1.67	0.05	0.61	3.68
	30%	6.67	0.086	2.16	0.12	1.08	3.48
FBFA	Raw	5.48	8.64	35.12	4.47	6.47	26.35
	10%	6.23	0.95	1.24	0.06	0.83	1.54
	20%	6.18	1.34	2.41	0.13	0.66	2.16
	30%	6.03	2.41	3.05	0.20	1.64	2.67
GB 16889-2008			40	100	0.25	4.5	100

ND: < 0.2 × 10^−3^ (mg/L).

**Table 7:** The results of the leaching test according to USEPA SW-846 Method 1311.

MSWI-FA Type	Amount of Addition	pH Value	Heavy Metal				
			Cu	Zn	Pb	Cr	Ba
			Unit (mg/L)				
GFFA	Raw	12.14	0.198	4.05	3.51	1.94	4.81
	10%	10.51	N.D.	N.D.	N.D.	N.D.	5.61
	20%	10.53	N.D.	N.D.	0.0032	N.D.	5.12
	30%	10.56	N.D.	N.D.	0.0043	N.D.	5.84
FBFA	Raw	8.54	N.D.	N.D.	N.D.	N.D.	10.54
	10%	10.21	N.D.	N.D.	N.D.	N.D.	6.24
	20%	10.24	N.D.	N.D.	N.D.	N.D.	6.13
	30%	10.13	N.D.	N.D.	N.D.	N.D.	6.35
Regulatory Limits			--	--	5.0	5.0	100

ND: < 0.2 × 10^−3^ (mg/L).

**Table 8:** The results of the leaching test according to the USEPA SW-846 Method 1312.

MSWI-FA Type	Amount of Addition	pH Value	Heavy Metal				
			Cu	Zn	Pb	Cr	Ba
			Unit (mg/L)				
GFFA	Raw	12.29	0.184	5.47	3.51	1.84	6.84
	10%	12.12	0.0019	0.089	0.12	0.034	0.012
	20%	12.14	0.0039	0.243	0.14	0.051	0.021
	30%	12.18	0.0050	0.465	0.21	0.062	0.054
FBFA	Raw	12.16	0.743	6.84	4.47	1.24	8.25
	10%	11.94	0.059	0.037	0.51	0.018	0.0019
	20%	11.96	0.124	0.069	0.62	0.046	0.0029
	30%	11.94	0.236	0.085	0.72	0.052	0.0041

ND: < 0.2 × 10^−3^ (mg/L).
